# The British Journals: Surgery

**Published:** 1838-10

**Authors:** 


*Med. Gazette. July* 7, 1838.

### SURGERY.

*On the Primary Causes of Strangulation, and an improved Mode of performing the Taxis, in Cases of Intestinal Herniæ.* By James O'Beirne, M.D., one of the Surgeons to the Richmond Surgical Hospital, &c., Dublin.

[This is a most valuable practical paper and deserves the attention of all surgeons, although it will present but little novelty to those who are acquainted with the author's excellent work, published some years since, and entitled "New Views of the Process of Defecation." The present article, however, gives more detailed views of the principles and practice laid down in that work respecting the reduction of hernia, and contains, moreover, "the whole of the cases, published and unpublished, successful and unsuccessful, in which the practice has been employed during the last eight years." These cases are sixteen in number, and exhibit, according to Dr. O'Beirne, "positive proofs of this mode of treatment having obviated the necessity of an operation in eleven out of sixteen cases of complete strangulation, where the usual and most efficacious means had previously been resorted to in vain." The following extracts explain Dr. O'B.'s views of the disease, and his peculiar mode of practice.]

Having before us this view of the strangulated and other portions of the intestinal tube, we are enabled to see at once, that, in such cases, various forces oppose our attempts at the taxis, and that these forces are as follow, viz.: the great size of the hernial tumour, compared with that of the ring—its almost immoveable attachment to the latter—its great firmness and incompressibility, its enlargement above as well as below the stricture—*the constant current and pressure of flatus into the constricted and distended intestine*—the acute angles formed by the convolutions within the abdomen, and immediately above the stricture, and the extremely unyielding nature of the ring itself. The consideration of such numerous, various, and powerful forces acting in opposition to our attempts at the taxis, is well calculated to show the difficulties and uncertainty in effecting reduction; the injury likely to be done by using much force; and the little power which the hands, however judiciously applied, can exert over a tumour so situated. It is true, that notwithstanding the opposition of all these forces, such attempts have often proved successful; but I apprehend that in such instances, their success was more owing to an occurrence not observed or enquired into, and which I shall notice hereafter, than to any peculiar tact or manipulation on the part of the operator. But the question which we have now to consider is, how are all these forces and difficulties to be overcome? We see that they are all but the products of distention of the strangulated intestine by flatus, and we know that we can evacuate the flatus by dividing the stricture, or by puncturing or cutting into the intestine itself. But as our object is to avoid the first, while the two last can only be thought of in an argument, the very simple question suggests itself, can we, by any other means, empty the bowel of its gaseous contents? This naturally leads us to survey the state in which the whole of the intestinal canal is placed; and we find, first, that the cavity of the strangulated gut is not obliterated, but permits air to pass freely into and out of it; secondly, that all that portion of the small intestines within the abdomen, and the whole of the cæcum and colon, are dilated, and also afford free passage to air; thirdly, that the rectum is firmly contracted, and that it alone opposes the exit of this elastic fluid. The instant that we obtain this view of the facts, it becomes manifest, that the great object in view may be quickly obtained, merely by introducing a gum elastic tube through the rectum, into the colon, and retaining it in that situation until the flatus contained in the large and small intestines, and ultimately that in the strangulated intestine, is completely evacuated, and the tumour so diminished in size and tension, as to start up into the abdomen, as it often does, *proprio motu*, or be easily reduced by the taxis. But to complete this view, it is necessary to consider the circumstances which may occur, either to cause the complete failure of this mode of assisting the taxis, or merely defeat the first attempt at its employment, and afterwards prevent its complete success. If the strangulated bowel be rendered impervious by any of the causes which I have already mentioned, it is clear that the introduction of the tube cannot possibly have the effect of emptying the hernial tumour; but, as the existence of these causes cannot be ascertained beforehand, the fact only shows the propriety of employing the practice in all cases indiscriminately. If, previous to, or during strangulation, the bowels happen to be loaded with solid or fluid fæces, the lodgements of fæcal matter in the cæcum, the lower portion of the ileum, and the sigmoid flexure of the colon, will necessarily prevent the passage of flatus, and prevent the success of the first introduction of the tube; but this circumstance only shows the necessity of persevering in the practice, assisted by repeated enemata, in order to eventually succeed.

But let this amount of success (eleven cures out of sixteen cases) be calculated or viewed how it may, it is manifestly such as ought to compel every conscientious and unprejudiced surgeon to give the practice a full and fair trial, before he decides on an operation which too frequently terminates fatally, and requires for its performance a degree of skill which many do not possess, and able assistance which may not be at hand. Such a trial causes very little loss of time, does not interfere with the employment of other remedies, is perfectly safe, and may be had recourse to after reasonable attempts at reduction have failed. For the reasons already stated, I have advised the treatment to be employed indiscriminately in all cases of strangulated intestinal hernia; but there is an exception to this general rule, namely, the cases in which the hernial tumour is inelastic at all points, and evidently impacted with hardened excrement which cannot be forced into the bowels within the abdomen. The cases in which its effects are most valuable and striking, are those of small, recent, and strangulated enterocele, in which the constitutional disturbance is so severe and urgent, and the hernial tumour so tender, that the patient will not allow it to be touched, or cannot bear any attempts at the taxis. In cases where the intestine adheres to the sac, although it cannot effect reduction, it may either remove or relieve the symptoms of strangulation. But as far as my experience goes, it is as inapplicable in practice as it is in theory to cases of epiplocele.

In my work on Defecation, I have described the different tubes and syringes which I had employed from time to time, in diseases of the intestinal canal; but, from some strange oversight, have omitted to describe the most improved; and as they are those which I continue to employ in all such cases, it is necessary to supply this serious defect. The gum elastic tube is sixteen inches in length; considerably thicker throughout, and more bulbous at its upper extremity than that of the stomach-pump; at its lower extremity, it has a brass ferule, so made as to fit bayonet-wise into an aperture in a short pipe springing from a small brass syringe; and to give it the necessary firmness, a delicate brass wire runs spirally through its interior. I employ this tube in new-born infants, as well as in adults. The syringe which I use is of brass, about seven inches long, and one inch in diameter, it is easily worked, very portable, and quite sufficient for all purposes connected with intestinal affections. In short, it is the small syringe belonging to the self-injecting apparatus of Mr. Weiss of London, to whose ingenuity I am also indebted for the necessary improvements in the gum elastic tube.

Having known that many intelligent and expert practitioners have failed in introducing the tube to the necessary height, and that others are fearful of using the degree of force sometimes necessary for that purpose, I may be permitted to offer a few simple instructions to the one, and some facts and reasoning to the consideration of the other. As regards the difficulty of introducing the tube to the necessary height, it arises from the non-observance of the following rules. 1st. The instrument should be thrown into cold water until it becomes stiff, then be dried, made perfectly straight, and a few inches of its upper extremity well oiled. It is then to be introduced, the patient lying on the left side, and passed up inch by inch, and as nearly as possible in the course of the rectum. If obstruction be met with, it may be slightly withdrawn, and afterwards passed gently upwards. But if it cannot be introduced higher up without much force, attach to it the syringe, and dipping the point of the latter into the fluid to be injected, let an assistant give two or three rapid strokes of the piston, and so as to bring the full force of a strong and unbroken column of fluid to bear upon the point of resistance, and, while this is doing, let the surgeon urge the tube firmly upwards, and it will generally pass through the obstruction, as if through a narrow ring. 2dly. With respect to the fear of using the moderate degree of force necessary to pass the tube into the colon, it is chiefly attributable to the fatal consequences which are known to have attended the unskilful management of rectum bougies, and the rude introduction of the metallic pipe of the common enema syringe. But the cases are quite different, and I shall now contrast them. The metallic pipe is some inches in length, and quite inflexible; the rectum bougie is short, thick, solid, and possesses little flexibility. With either of these, it is obvious that great injury may be inflicted on the bowel. The gum elastic instrument, on the contrary, is much longer than either, not so thick as the latter, tubular, bulbous at the upper part, flexible to the necessary degree, and, consequently, most unfitted for exerting any great or dangerous degree of force. Besides, its action must be that of dilating, not piercing, the sides or walls of the contracted rectum, for it is grasped, throughout four-fifths of its course, by the muscular coats of the intestine, and compelled to move in the axis of the bowel, and in no other direction. All these points should be well weighed first, and then viewed in connexion with the well known fact, that it requires very great force to rupture living muscular fibre. But the argument which ought to have most weight with those who fear to use the necessary degree of force, is that which I have used in my work, and which increased experience enables me to repeat, namely, the fact, that I have neither met with, nor heard of, a single instance in which the introduction of the gum elastic tube has been followed by an evil or unpleasant consequence.

*Dublin Journal of Med. Science. September* 1, 1838.

---

*Observations arising out of the Results of Amputation in different Countries.* By BENJ. PHILLIPS, Esq. F.R.S., Surgeon to the St. Marylebone Infirmary.

[THIS paper was read before the Med.-Chirurgical Society in November last. It is a very valuable document, and well calculated to promote the great object of substituting, in medicine, logical deductions founded on positive facts, in place of traditional statements derived from vague suppositions. We regret that our limits will not permit us to lay before our readers the excellent introductory observations of Mr. Phillips: we must, however, find room for the more important facts established by his investigations.]

The amputations included in this enquiry are those of the arm and the forearm, the thigh and the leg. The whole of them have been performed within the last four years in civil hospitals and in the private practice of hospital surgeons. The gross number of cases is 640; and this number embraces all cases, acute, chronic, and the results of violence which have occurred in the practice of the persons by whom the returns have been furnished within the period I have named. Of these cases, 490 are reported "cured," and 150 died, either in consequence of the operation or the progress of the disease; to rescue the patient from which, recourse was had to the operation.

I apprehend that a large number of our professional brethren are unprepared for such a result: I have only met with very few who were at all sensible of the extent of the mortality which occurs. Compared with lithotomy, amputation and the ligature of arteries are often, perhaps commonly, held to be unimportant operations; and yet the results show a very great balance in favour of the success of lithotomy. It is necessary, however, in connexion with this circumstance, that a fact should be borne in mind, by which these results will be modified. A large number of amputations are performed under very unfavorable circumstances. Lithotomy, on the contrary, commonly admits of our choosing a time when the patient is best prepared for it. In other words, the mortality consequent upon lithotomy is often directly caused by the operation: in amputation, by the disease or injury.

I have now shown that the mortality succeeding to amputation is very great,—twenty-three per cent. I shall, therefore, proceed to analyze the gross number, and exhibit the proportion furnished by the different countries implicated in the enquiry. They are as follow:

| | Cases. | | Deaths. | | Per Cent. |
|---|---|---|---|---|---|
| France | 203 | ... | 47 | or | $23\frac{31}{203}$ |
| Germany | 109 | ... | 26 | ... | $23\frac{93}{109}$ |
| America | 95 | ... | 24 | ... | $25\frac{5}{19}$ |
| Great Britain | 233 | ... | 53 | ... | $22\frac{174}{233}$ |
| | 640 | | 150 | | $23\frac{7}{16}$ |

Here is an average number of deaths, amounting to, as near as may be, 23½ per cent. If the several countries be taken separately, we find that France is a fraction below this average; that Germany differs only to the amount of a fraction from France; that America only exceeds the average by a little more than two per cent.; and that Great Britain is a fraction below the average.

. . . .

We now proceed to enquire whether the treatment commonly, and in this country almost universally, employed after amputation in diseases, attended by proper suppuration, be fortunate in its results; and whether another method of treatment might not as a rule be advantageously adopted.

I have classed the observations which I possess, so as to show, on the one hand, the cases in which amputation has been performed in acute diseases, or in consequence of injuries; and, on the other, the cases in which it has been employed as a remedy for chronic diseases: but, as chronic disease is a very general term, I will point out the diseases which I have included in it; they are diseased articulations, with considerable purulent discharges, necrosis, caries, and extensive old-standing suppurating surfaces. In our own country, for a considerable number of years, the method of immediate union has been almost exclusively employed. No matter what may have been the disease, it has commonly been attempted. The inconveniences of secondary union were doubtless considerable; so that the system of immediate union, once adopted, should have been exclusively employed, was a result very much in conformity with the revolutions of all systems.

The idea of lessening pain by diminishing the number of dressings, of avoiding profuse suppuration, of rapidly healing a large wound, were powerful elements for the overthrow of the old method. At the same time, its brilliant success in the practice of military surgeons came so strongly in support of the new system, that the abandonment of the system of consecutive union in our own country was, and still is, almost complete. In other countries, the merits of the new system were much less readily appreciated, and the prejudice which was raised against it was considerable. Men clung with pertinacity to the old system, disastrous as were its effects; and, even up to the present moment, many eminent surgeons employ it almost exclusively,—modified, however, it is true; and a very large number employ it much more extensively than we do.

. . . .

I beg attention to the results which I shall now proceed to lay before the Society, in proof of the opinion that there is a large and well-defined class of diseases in which, after amputation, union by the first intention cannot, as at present practised, be so successfully employed as that in which it is consecutively obtained.

This class of diseases I have already particularized; and, of the 640 constituting the gross number on our tables, 213 cases are of this kind.

Of these cases, immediate union was attempted in 117; consecutive union in 96.

Of the 117 cases, 88 only succeeded; the deaths amounted to 29.

Of the 96 cases in which the treatment was by consecutive union, 76 succeeded; the deaths were 20.

Of these cases, Great Britain furnished 86; the other countries included in the observations, 127.

Of the 86 cases, immediate union was attempted in 60; consecutive in 26, and with the following result:—Of the 60 cases, there were 15 deaths; of the 26, there were 5 deaths.

Of the 127 cases, immediate union was attempted in 57 cases; consecutive in 70.

Of the 57 cases, 14 were unsuccessful, (the patients died;) and 43 succeeded. Of the 70 cases where consecutive union was employed, there were 15 deaths. The results, therefore, attendant upon the practice of immediate union are, a mortality amounting to 25 per cent.; upon consecutive union, of nearly 21 per cent.

And there is a singular uniformity attendant upon the results of these two modes of practice, as shown by the returns furnished by our own and the other countries; and all are strongly confirmatory of the prudence of avoiding immediate union in this large and well-defined class of diseases.

As, I trust, this has been made sufficiently evident, it may now be asked, in what way is this increased fatality, which is attendant upon immediate union, to be explained? With respect to a portion of the cases, the explanation is easy:—they have been produced by phlebitis and purulent absorption; pus being found in the lungs, liver, and other situations. The reason why, in such cases, the tendency to this termination is more frequent than in ordinary cases is, I apprehend, because the disposition to purulent secretion in the diseased organ still continues; and because immediate union might prevent its evacuation, and so cause constitutional disturbance and absorption. In a certain number of cases, "visceral congestion" is occasioned by the suppression of the accustomed secretion; diarrhœa supervenes, and the patient dies. . . .

Having indicated the result, and being strongly impressed with its correctness; having adduced evidence which militates strongly against the practice of immediate union in the class of cases to which I have before particularly alluded; it may be asked whether I have any suggestion to make, by the adoption of which the inconvenience of the present practice may be lessened? . .

I am not an advocate for the substitution of consecutive for immediate union in any class of cases, but I am prepared to recommend a modification of the present method of treatment in a large class. If the causes of the adverse results of immediate union have been correctly indicated, I apprehend that a means of remedying them will be naturally presented. If it be a consequence of the sudden suppression of a secretion to which the economy has been long accustomed, we may fairly assume that, if the secretion can be maintained during a certain variable period of time by artificial means, we may employ the method of immediate union without any apprehension of those complications to which allusion has been made, and at the same time avoid those objections of which consecutive union cannot be relieved. Virtually, the proposition is carried into effect every day. An attempt is made to secure immediate union in such cases: it is unsuccessful; consecutive union obtains; the secretion is indefinitely prolonged, but the evils of consecutive union come into play.

My proposition is stated in a few words. Before the amputation is performed, establish in the vicinity of the point where amputation is to be practised an artificial suppurating surface, either by means of a seton or issue, and let the secretion be maintained for a term varying with the duration of the secretion at the diseased point.

[Mr. Phillips admits that he has no direct evidence of the efficacy of the proposed modification of the practice; but he considers himself fully borne out by the facts adduced, and by legitimate reasoning, to recommend it to the profession.]

*Med. Gazette. June* 9, 1838.

---

### *On the Treatment of Small-pox by puncturing the Pustules.*
By JOHN LANGLEY, Esq.

[ALTHOUGH the treatment recommended in the following extract is by no means new, it is believed to be less known, and still less practised, than it ought to be.]

At the present time, when I regret to observe in this district that that baneful disease, small-pox, has been and is still existing to a considerable extent, any mode of practice which has for its object and effect the amelioration of suffering, &c. ought to be freely promulgated. Since my publication of the following mode of practice, very many cases of small-pox have fallen under my treatment; and of those in which the pustules have by puncture and incision been evacuated, I have every reason to be satisfied that the desirable objects above alluded to have been invariably attained. In almost every case of small-pox where, from the malignity of its character, height of attendant inflammatory fever, and confluent nature of eruption, serious consequences are to be apprehended, upon the pustules acquiring a full and prominent vesicular character, before the lymph assumes its purulent appearance, I puncture with a lancet the most depending part of the inferior edge of the pustule; thereby leaving a perfect superficial covering of cuticle, obviating in a great degree the tendency to irritation resulting from the ingress of atmospheric air into the collapsed cyst, the superjacent cuticle falling as a plaster upon the secreting surface. The expression of relief afforded to the sufferer by this operation is most grateful, and may be somewhat adequately estimated by those who have experienced the comforts of having the contents of a whitlow evacuated. The desquamating process is not only facilitated, but greatly expedited; as, in the course of three or four days, you have an extensive desquamation, occurring almost simultaneously over the whole surface, leaving discoloration, but seldom any depressions: and in cases where, for the sake of experiment, I have thus proceeded with one superior extremity, and neglected the other, the difference of result has been most manifest. The removal of the distressing tension pervading so extensive a surface, and pressing upon such sensitive and irritable tissues, cannot fail to give the *rationale* of the relief afforded, and also the fair presumption that the cerebral irritation, and consequent fever resulting from such a painful condition, must be greatly modified, if not altogether obviated, independently of removing from the inflamed and abraded surfaces an immense quantity of morbific secretion, the reabsorption of which, to a certain extent, may be reasonably inferred. If, from a prejudice, which oft ignorantly prevails, of interfering with the natural course of naturally incidental diseases, certain persons might not grant the propriety of opening all the cysts, (which, by the bye, is no trifling task,) surely they will make trial of the practice upon those parts, the preservation of which is so desirable. *Lancet. June* 30, 1838.

---

*On the Efficacy of Pressure in certain Cases of Venereal Phagedænic Ulceration.* By Hugh Carmichael, a.m., Member of the Royal College of Surgeons in Ireland.

[This is a valuable practical paper, and recommends a mode of treatment, in a most untractable affection, which we doubt not will be found frequently, if not generally, beneficial. Surgeons who have been in the habit of witnessing the excellent effects of pressure in cases of inflamed testicle, erysipelas, and ulcers of the legs, will have no difficulty in admitting the probability of advantage from the extension of the practice to phagedenic syphilitic ulcers. The following extract from Mr. Carmichael's paper contains the general views of the author, and an account of the mode of applying the pressure: they are illustrated by the detail of four cases.]

Great irritability being one of the most prominent features of the disease, I was induced to imagine that pressure (an agency used with so much benefit in ill-conditioned, unmanageable ulcers generally, where morbid sensibility is a very leading character,) might probably, in these, be likewise adopted with some advantage: it was accordingly tried in a case that occurred to me of the most hopeless description, where all the varieties of treatment now generally employed were resorted to without effect, the disease progressing, and rapidly destroying the part; and the success which attended it was so decided that I have since used it in several others, and with such benefit as to establish it, in my mind, as a method highly deserving of attention in these cases. The great obstacle I have experienced in its use is the occasional difficulty of its application, so as to bring the diseased part decidedly under its influence: by a little dexterity, however, we may in most instances succeed in doing so, the operation sometimes requiring more management than at others. The mode of affecting it, of course, will vary according to the part the ulcer to be compressed is situated on: when on the glands or body of the penis, strips of adhesive plaster are the means I have adopted, looped, by passing one of the tails through a slit in the other; the penis is then to be introduced into the loop, and, the ulcer being brought into its bearing, it may be tightened at pleasure; the tails are to be then firmly wound round the penis, and secured. When on other parts, as the forehead, or places similarly circumstanced, pressure may be more easily and decidedly commanded; while on others it may, perhaps, be more difficult to effect it; yet by management, I think, with very few exceptions, it can be accomplished in all. On some occasions, when the required pressure should be more decided, I have employed slips of sheet-lead, placed over any appropriate dressing, and included in the loop. This substance, from its pliant nature, admits of being easily moulded into any form, and can readily be shaped so as to produce effectual compression upon the ulcer. Indeed, I think that the beneficial effects to be derived from pressure, particularly in ulcers, is not so much from the degree of tightness with which it is used, as in the application of a solid, unyielding substance to the surface, probably thereby inducing the absorption and removal of such diseased surface; and, for the reasons just stated, sheet-lead I have found to answer best for that purpose. With respect to the time required for its continuance before its full effects were obtained, it was various: sometimes a few days changing the entire character of the ulcer, from an ill-conditioned, spreading sore, to one of a florid, healthy aspect, with contracting boundaries; while, on other occasions, it required a longer continuance; but in all the amendment was so evident after the second or third day, as decidedly to manifest its salutary influence, and give assurance of a favorable result. In some instances I have been enabled, by means of it alone, to perfect the healing of the ulcer, unaided by any other measures; while in others, (and these the greater number,) its phagedænic nature was only removed; a morbid diathesis still remaining, which appeared incapable of being overcome entirely without more active remedies. In these latter I found mercury to serve all the purposes required; the phagedænic character being first subdued, the regenerated sore rapidly disappearing under its influence, when the system became engaged by it: indeed, it would seem as if its use were necessary in them to complete the cure. In the cases in which I employed it no other means were adopted, and in all it was successful,—I mean so far as subduing the phagedænic disposition: some, however, no doubt, may occur where it could not be used alone or in the first instance; for example, if great inflammation were present, leeching, with a view to the subduing it, according to the suggestions of Mr. Richard Carmichael, who has advised local bleeding in these cases, would, I think, first be necessary; and other circumstances might also be attendant upon it, requiring appropriate remedies before submitting it to pressure: these, however, could only be regarded in the light of preparatory steps, previous to the employment of this latter measure, and which must be considered that whereby effectual benefit is to be obtained: perhaps there may be cases where it would be productive of no advantage, or altogether inadmissible. *Dublin Journal. September*, 1838.

---

*On Chronic Cystitis; with Observations on the Employment of the solid Nitrate of Silver in Catarrh of the Bladder, as practised by* Professor LALLEMAND, *of Montpellier.* By JOHN O'BRYEN, M.D. M.R.C.S.

[THIS paper was read before the Bristol Medical Library Society, and is a valuable communication, inasmuch as it will make more known to the profession in this country a considerable improvement in the treatment of a common and very distressing disease.]

If you reflect (says Dr. O'Bryen,) upon the severity of chronic cystitis, which irresistibly compels a man to get out of bed four or five times per hour during the night, each time subjecting him to excruciating pain, and thus effectually preventing sleep, and which will not permit him to sit one half-hour at his dinner-table without disturbing him; to say nothing of the effect of pain on the general health and the constitution, or the want of exercise on horseback or in a carriage, or even on foot; and then again reflect that, when of long standing, it has been declared incurable, inveterate, and the opprobrium of surgery, though it may be relieved, but, like chronic bronchitis, returns with redoubled severity upon the application of the slightest exciting cause; I feel assured you will agree with me that any mode of treatment, offering for such a disease not relief alone, but a radical cure,

—and that, too, not in months, but weeks, and often in a few days,—deserves our best attention, and its author our meed of praise.

The following is Professor Lallemand's mode of applying the remedy:

He uses an instrument which consists of a large catheter (of pure silver, as, if there is any alloy, the caustic acts upon it,) open at both ends, having two sorts of stilet, according to the part you wish to cauterize; at the extremity of each stilet is a small excavation, containing the caustic, which is first pulverized, and then placed in the excavation over a spirit-lamp, which fuses and moulds it to the cavity. When the instrument is prepared, introduce into the bladder an ordinary catheter, in order to empty it completely. This precaution is strictly necessary, for the urine would dissolve the caustic, and prevent its directly affecting the mucous membrane. When this has been withdrawn, the instrument bearing the caustic is to be introduced (closed), and the moment it has entered the bladder, you are to push the stilet, and rapidly turn the *porte caustique* from side to side two or three times, and then pull the stilet into the instrument, and withdraw it: our object should be to touch the surface in as many points as possible. While the instrument is within, the bladder contracts and grasps it, the kidneys secrete a small quantity of urine, as the lachrymal gland secretes tears when the conjunctiva is cauterized; but this small quantity of liquid, far from being hurtful, is, on the contrary, favorable, as it acts as a vehicle to the portion it does not decompose, and conveys it equally over the surface of the membrane. The patients feel at that moment a sharp pain at the neck of the bladder and in the rectum, described by them as not unlike a pinch, but much more supportable than the continued dull pain of chronic catarrh. There is now an irresistible desire to pass water, and, as the bladder is nearly empty, very little passes; and this causes a burning along the urethra, and is accompanied by some drops of blood. This desire is renewed every moment, causing violent but useless efforts. These gradually decrease; and on the second and third day there is no longer any pain on making water, and a few small grey eschars, like burned paper, come away with the urine. This occurs in a large number of patients; but in some more susceptible the process does not proceed so simply, particularly if you have used the *porte caustique* too long. In this case retention of urine follows, which lasts from three to thirty-six hours: even here we must not be in too great a hurry to use the catheter, as a warm bath, a few narcotic lavements, emollient drinks, some tartrate of soda with infus. sennæ, and sometimes a few leeches, will cause the spasms to yield: if not, some belladonna to the meatus may be tried, always taking care to use antiphlogistics with moderation in the beginning, as inflammation is necessary to the cure. In a majority of cases, one cauterization is sufficient to procure a cure: when it happens otherwise, a second and even a third application may be necessary, but Monsieur L. states that he never saw a case requiring a fourth.

[According to Dr. O'Bryen, the advantages of this mode of practice are very great, when cases suited for it are selected: it is always inadmissible when the kidneys or prostate are diseased.]

Compare this with the modes of treatment hitherto employed: with what perseverance must they not be continued in for months, nay, often for life, to procure even relief. Patients treated as formerly must use great precaution to prevent a relapse: the least fault in diet, the slightest cold or change in the temperature of the air, the most moderate exercise on horseback or in a carriage, may act as a match fires a train, and make the sufferer as bad as ever: in fact, they must be always on their guard, even to enjoy a supportable state of health. This is not the case with cauterization; for, however feeble the digestive organs are, however weak the patient, whether in health or constitution, it may be employed with advantage, as it is never injurious: it acts directly on the diseased surface, and does not affect the other viscera; and the patients, instead of being months and years under treatment to procure even a slight amelioration, may be cured in a few days, or at latest in a few weeks. The cure is not an uncertain one, and the patient may return to his ordinary mode of life without fear.

*Dublin Journal. September,* 1838.

